# Comparative study on measurements of radiochromic films using portable colorimeters

**DOI:** 10.1038/s41598-024-54017-9

**Published:** 2024-02-09

**Authors:** Hiroshi Yasuda, Shido Morioka

**Affiliations:** 1 Research Institute for Radiation Biology and Medicine (RIRBM), Hiroshima University, Kasumi 1-2-3, Minami-ku, Hiroshima, 734-8553 Japan; 2 School of Medicine, Hiroshima University, Kasumi 1-2-3 Minami-ku, Hiroshima, 734-8553 Japan

**Keywords:** Biophysics, Environmental sciences, Engineering

## Abstract

The recently proposed method for on-site radiation dosimetry by a combination of radiochromic film and portable colorimeter was tested for six combinations of two popular Gafchromic films (EBT3 and EBT-XD) and three commercially available portable colorimeters (nix pro2, nix spectro2 and Spectro1 Pro; abbreviated to “NixP”, “NixS” and “SpoP”, respectively). EBT3 and EBT-XD were irradiated with X-rays (160 kV, 6.3 mA) up to 40 Gy and 80 Gy, respectively, and the radiation-induced color levels of RGB and CMYK components were measured with the three colorimeters. Angle dependence was examined by reading at 15° intervals. As a result, it was judged that all combinations would work effectively under certain irradiation conditions. NixP and NixS were applicable to a wider dose range for both films, while SpoP fit a lower dose range. On the other hand, SpoP showed an advantageous feature of no angular dependence in reading films, while NixP and NixS showed significant angle-dependent changes. These differences are considered to be attributable to the different geometries of LED light emission, which came from all directions (360°) in SpoP, 4 directions in NixP, and 8 directions in NixS. These findings are expected to expand the applicability of the novel method to various occasions of on-site dosimetry.

## Introduction

The first commercial product of polydiacetylene-based radiochromic film, called ‘Gafchromic’ (International Specialty Products, Wayne, NJ, USA) appeared in the late 1980s for industrial use^1^. While this initial product had a good dose–response feature, it had some issues regarding sensitivity, uniformity and reproducibility in the development of color images^2^. After continuous efforts to improve the dosimetric properties, recent products (Gafchromic EBT series) of which the first model was commercially released in 2004 achieved higher spatial resolution, weaker energy dependence, and less insensitivity to visible light in a normal indoor condition^3–9^. With these advanced features and its reasonable cost, Gafchromic films have been gaining popularity for quality assurance (QA) of external radiotherapy beams^10–17^. Additionally, in recent studies, the application of Gafchromic films to dose verification at ultrahigh dose rates (> 40 Gy s^−1^) in FLASH radiotherapy has been investigated^18–20^.

The advanced features of the recently developed Gafchromic films such as self-developing visible color change immediately after radiation exposure and long-term stability of the coloration level under normal environmental conditions have good potential for application in many fields other than dose verification for delivering radiotherapy beams. As one of such potential applications, the recent robust, light-insensitive products of radiochromic film are expected to be effectively used for occupational radiation monitoring of workers who routinely handle high-level radioactivity materials or who are in charge of the response to nuclear/radiological emergencies, i.e., those who have potential risks of accidental high-dose radiation exposure. On these occasions, the feature of direct color change immediately after significant radiation exposure that can be detected by the naked eye should be useful for earlier recognition of the occurrence of an unexpected radiological accident, which will enable us to swiftly identify severely exposed patients and then take necessary medical actions to save their lives without delays.

It should be noted that, while real-time individual monitoring can be made with a portable electronic personal dosimeter (EPD), EPDs hardly work in general owing to the count-loss problem experienced in the common situation of exposure at a high dose rate (e.g., several Gy min^−1^) in a radiological accident. Though this count-loss problem can be averted by employment of passive dosimeters or some artificial/natural materials that stably hold radiation-induced free radicals^21–24^, these techniques based on the readings of thermally/optically stimulated luminescence or electron paramagnetic/spin resonance signals from the samples need large, nonportable equipment for reading, and thus we could hardly detect an accidental exposure and perform the prompt dose assessment on site. Some approaches of biological dosimetry using human tissues such as tooth enamel and peripheral blood have issues that they need at least a few days for processing the tissue samples in a dedicated laboratory space; it would need a careful task for sample-specific dose calibration^25,26^.

With these thoughts, the authors recently proposed a novel method using a combination of a Gafchromic film and a portable dosimeter for on-site dosimetry^27^. This method, which skips the elaborate process for image acquisition of a radiochromic film using a flatbed scanner followed by subsequent analyses with an image processing tool, would widely expand the potential of radiochromic films to various occasions where on-site dosimetry would be needed. In the present study, the authors try to present the effectiveness of this method by testing several combinations of two Gafchromic films and three portable colorimeters that are commercially available at present.

## Materials and methods

### Materials employed

Two Gafchromic films, EBT3 and EBT-XD (International Specialty Products, Wayne, NJ, USA), were employed as radiochromic materials. Both films are currently popular as recent products of the EBT series, which appeared in 2004. The newest EBT-XD film has some improved features over EBT3 for dosimetry of external radiotherapy beams. Both films have similar physicochemical properties^8,13,14,28,29^, such as a thin active layer (28 μm in EBT3 and 25 μm in EBT-XD) sandwiched with 125 μm-thick polyester substrates; the active layer is made of crystals of lithium-10,12-pentacosdiynoate (LiPCDA) compound with a diameter of 1 to 2 μm, marker dye, and other stabilizers and additives. A notable difference is the applicable dose range; the optimal dose range of EBT3 is 0.1–10 Gy, and the dynamic dose range is 0.1–20 Gy, while that of EBT-XD is 0.4–40 Gy and 0.1–60 Gy, respectively^28,29^. The LiPCDA crystals of EBT-XD are shorter in length (2–4 μm) than those of EBT3 (15–25 μm); the small-size active compounds work to reduce the unfavorable polarization and light scattering; this would result in fewer lateral artifacts, so-called ‘orientation effects’, which have been observed as angle dependencies in the color digitization process of Gafchromic films scanned with flatbed scanners^7–9,30–32^.

The color intensities of Gafchromic films were measured by using portable colorimeters with the aim of achieving practical on-site dosimetry that should be beneficial for the prompt response to an unexpected event of accidental high-dose exposure. Three commercially available portable colorimeters were employed here: “nix pro 2” (Nix Sensor Ltd., Ontario, Canada; ab-breviated to “NixP” hereafter), “nix spectro 2” (Nix Sensor Ltd.; abbreviated to “NixS”), and “Spectro1 Pro”, Variable, Inc., Tennessee, USA; abbreviated to “SpoP”). These products contain rechargeable lithium-ion batteries and can perform 100 scans or more per single charge.

**Figure 1:**
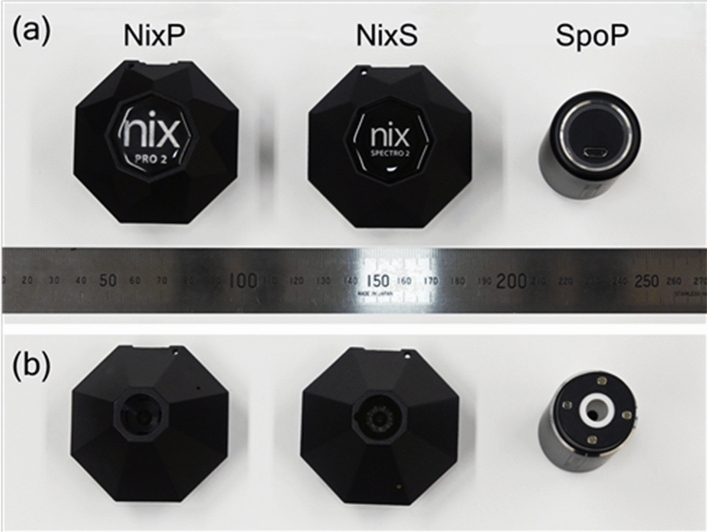
The sensors of three portable colorimeters employed in the present study. From the left, nix pro 2 (Nix Sensor Ltd.; abbreviated to “NixP”), nix spectro 2 (Nix Sensor Ltd.; “NixS”), and Spectro 1 Pro (Variable Inc.; “SpoP”). The upper photograph (**a**) was taken from the above and the lower one (**b**) taken from the bottom.

NixP and NixS have hexagonal shapes, and SpoP is cylindrical, as shown in Fig. 1a. All of them can measure the color intensities of any flat material while cutting the ambient light as color levels of RGB and CMYK components in a few seconds, and the measured data are transferred via Bluetooth to a smartphone installed with the exclusive application of each colorimeter and immediately displayed on screen. These data can be saved into the memory of an Android or iOS smartphone and exported to other devices for further analyses.

It should be noted that this method is not for two-dimensional dosimetry, for which radiochromic films have been generally used, since the aperture size of any portable colorimeter for detecting the light reflected from a film surface is more than 5 mm in diameter (Fig. 1b). Thus, for the purpose of multidimensional dosimetry, the conventional reading method using a flatbed scanner is necessary.

The high performances of these portable colorimeters in reading color intensities of RBG components has been presented in previous studies aiming to determine soil compositions^33–35^ and evaluate meat freshness^36–38^. The advantageous features of these products, such as small scale, light weight, easy handling and lower price, are favorable for wide application to on-site dosimetry on various occasions. Particularly, its excellent portability is beneficial for the aim of radiological emergency preparedness, since anyone can carry this device in a bag or pocket and use it promptly after the occurrence of an unexpected radiological event.

### Irradiation and film analyses

Both Gafchromic films (EBT3 and EBT-XD) were irradiated with static X-rays (160 kV, 6.3 mA) using a cabinet-type X-ray irradiation system (“CP-160”, Faxitron, Wheeling, IL, USA). Different doses were given to two films with consideration of their dynamic dose range: 0.3, 0.6, 0.9, 1.2, 1.6, 2, 3, 4, 5, 6, 8, 13, 20, 30, and 40 Gy for EBT3 and 1, 2, 4, 7, 10, 15, 20, 25, 30, 40, 60, and 80 Gy for EBT-XD. The X-ray dose rates delivered to the films were verified with commercially available radiophotoluminescence dosimeter (GD-352M, AGC Techno Glass Co., Ltd., Shizuoka, Tokyo). In these irradiations, we adjusted the dose rate of X-rays by changing the distance between the source and the film so that the irradiation time would exceed 2 min in every case. The diameter of the irradiation field was more than 5 cm even at the shortest distance.

An irradiated film was placed on a Kent paper (thick white paper commonly used for drawing) and measured with three portable colorimeters (NixP, NixS and SpoP). The data of color intensities as RGB (red, green and blue) and CMYK (cyan, magenta, yellow and black) components were acquired with an exclusive application provided by the company of each portable colorimeter through the Google Play store for use on an Android smartphone.

Considering the possible orientation effects (lateral response artifacts) observed in the readings of Gafchromic films using flatbed scanners, we examined the angle dependence of the measured color intensity by rotating a film at 15° intervals in a clockwise direction. Analyses of the acquired data and productions of graphs including regression analyses were made using commercial data analysis software (IGOR Pro, Wave-Metrics, Inc., Portland, OR, USA).

## Results and discussion

### Dose responses of radiochromic reactions

In the field of radiotherapy, the color intensity of a Gafchromic film has been generally evaluated with the net optical density change (*netOD*), which is calculated as a logarithm of the ratio of a color-channel value of each pixel before irradiation to that after irradiation. As the applications for three portable colorimeters just show the color intensity values and do not have a function to calculate the *netOD*, a person who like to estimate the dose needs to conduct a calculation for converting the color intensity to the dose. The authors think this dose conversion process should be as simpler as possible without a complex function for use in urgent on-site dosimetry in radiological emergency. Therefore, the radiation-induced change in color intensity (Δ*I*) was calculated in simple ways from the values obtained with a portable colorimeter as follows.

For the RGB component:

1 $$\Delta I = I\left( 0 \right) - I\left( D \right)$$

For the CMYK component:

2 $$\Delta I = I\left( D \right) - I\left( 0 \right)$$

where *I*(*D*) is the measured color intensity of the respective channel at dose *D* and *I*(0) is the color intensity measured just before irradiation. Because the film darkening caused by radiochromic reaction generally decreases the color intensities of the former and generally increases those of the latter, Δ*I* was calculated in the opposite way for the RGB and CMYK components so that major color components would generally present positive values.

**Figure 2:**
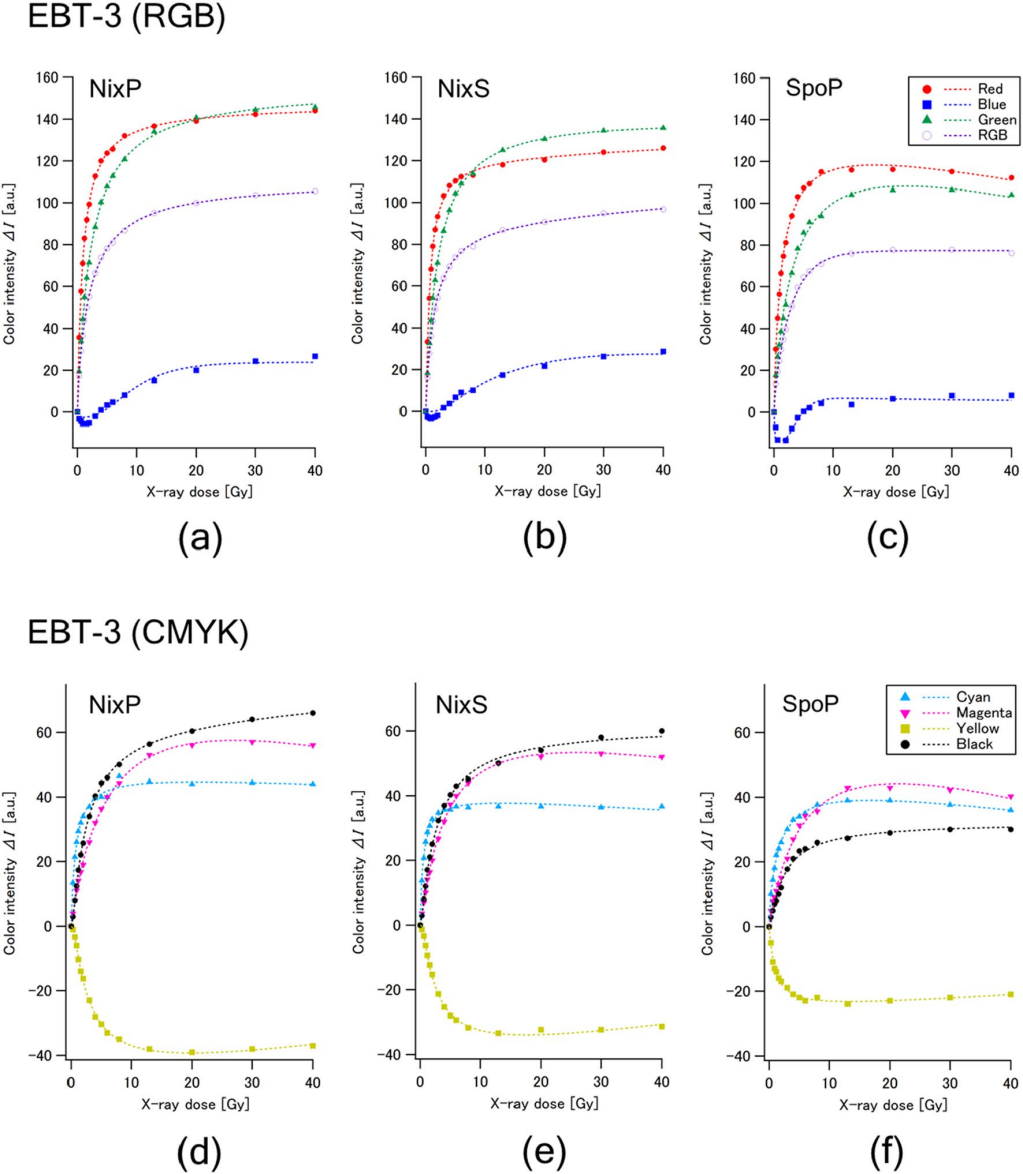
Dose responses of the color intensities of Gafchromic EBT3 film irradiated with X-rays at up to 40 Gy in regard to the RGB components (above) and CMYK color components (below) measured with three portable colorimeters: NixP (**a**, **d**), NixS (**b**, **e**) and SpoP (**c**, **f**) at the angle of 0°. The standard deviation of each plot was less than 3%.

**Figure 3:**
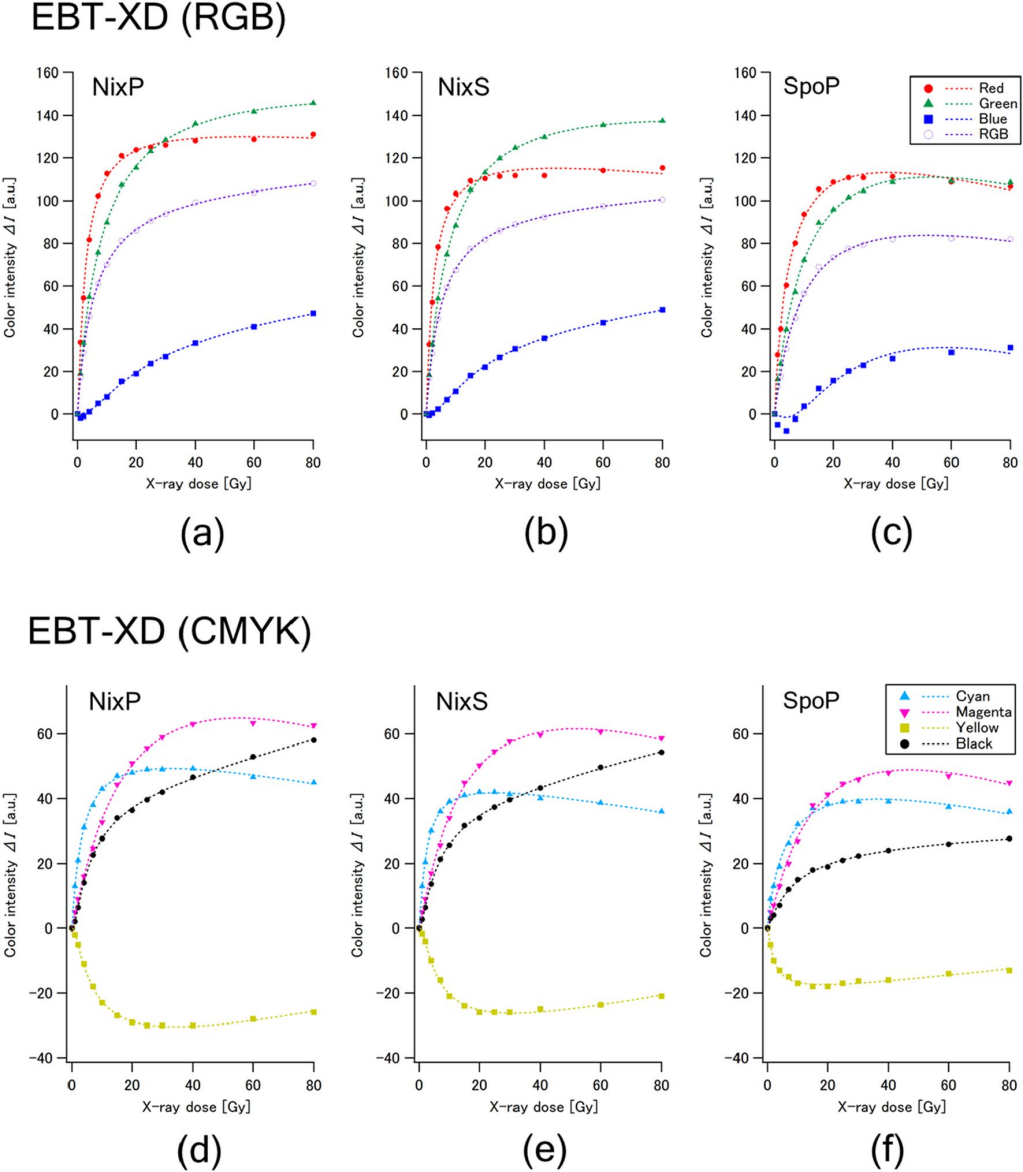
Dose responses of the color intensities of Gafchromic EBT-XD film irradiated with X-rays at up to 80 Gy in regard to the RGB components (above) and CMYK color components (below) measured with three portable colorimeters: NixP (**a**, **d**), NixS (**b**, **e**) and SpoP (**c**, **f**) at the angle of 0°. The standard deviation of each plot was less than 3%.

Figures 2 and 3 show the dose responses of color intensities of EBT3 and EBT-XD, respectively. The standard deviations of measured color intensities were less than 3% in all the cases. The blue component was less sensitive in every case and became negative in the lower dose range. The yellow component showed peculiar radiochromic reactions that were nearly opposite to those of other components (cyan, magenta and black). Accordingly, these two components were judged to be unsuitable for use in dosimetry. The red and green components presented advantages of higher sensitivity compared to the RGB component, which is a mixture of three components (red, blue and green).

Regarding EBT3 (Fig. 2), the red and green components showed comparable, simply increased dose responses in NixP and NixS. The red component increased more steeply than the green component, while the green component continued increasing at higher dose ranges. On the other hand, both components of SpoP decreased at higher dose ranges (> 10 Gy for the red component and > 20 Gy for the green component). The cyan component reached almost constant values at several to 10 Gy, and the magenta component decreased gradually at higher dose ranges (> 10–30 Gy). The intensity of black component simply increased with dose in all colorimeters; its sensitivity was the highest among those of the CMYK components in NixP and NixS, while the intensity of black component in SpoP was lower than those of the cyan and magenta components.

The dose responses of EBT-XD (Fig. 3) were similar to those of EBT3, while some different characteristics were observed. It should be noted that the color intensities of the black component were lower than those of the magenta component over the dose range up to 80 Gy in any colorimeter, while they showed a monotonic increase with increasing dose in every case. According to these findings, we judged that the use of a black (K) component is the most preferable for on-site dosimetry covering a wider dose range.

To discuss the applicability of each combination for on-site dosimetry, we examined the relationship between the X-ray absorbed doses as an objective variable and the black color intensity of Gafchromic film as an explanatory variable within their optimal dose ranges. Through careful investigations on the fitness of various formulas to experimental data, we decided to employ the following function composed of a linear term and an exponential term for regression:

3 $$\Delta I = a \times \Delta I + b \times {\text{e}}^{c \times \Delta I}$$

where *a*, *b* and c are the parameters for fitting that are empirically determined through regression analyses.

**Figure 4:**
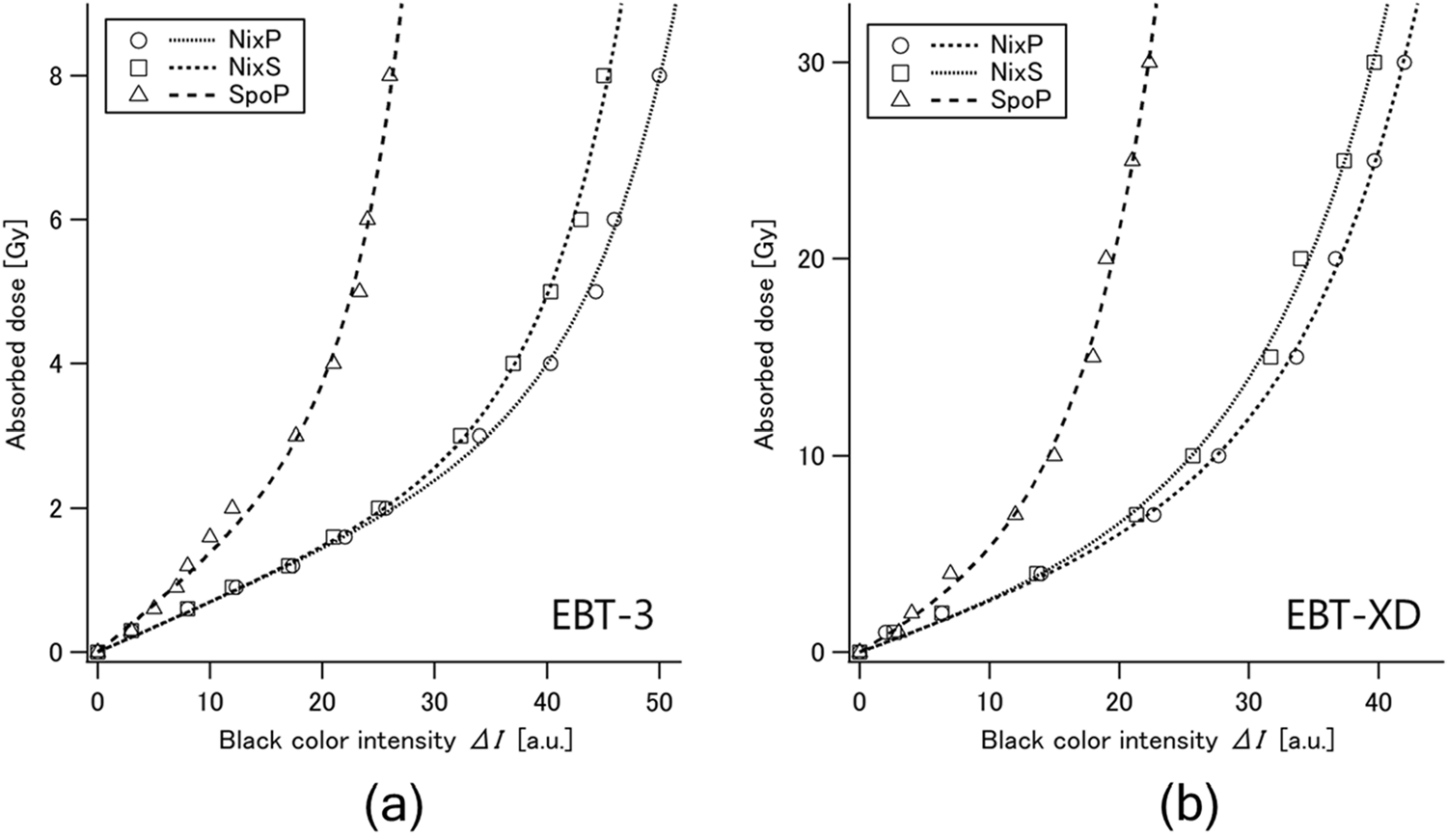
Comparison of the relationships between the color intensities of the black (K) component measured with three colorimeters (NixP, NixS and SpoP) for EBT3 (**a**) and EBT-XD (**b**) after X-ray irradiation at up to 8 Gy and up to 30 Gy, respectively. The standard deviation of each plot was less than 3%.

As seen in Fig. 4, the above function (Eq. 3) fit the experimental data of the black component well in all cases. The largest discrepancy between the measured and estimated doses was observed at the black color intensity of around 10 for SpoP, resulting in about 10% difference in relative error and about 0.2 Gy difference in absolute dose value. No differences greater than 0.2 Gy were observed in any cases. The curves of NixP and NixS were identical, while the curve of SpoP was much steeper. These findings indicate that SpoP is more suitable for a narrower, lower dose range than NixP and NixS. Values of the fitting parameters (*a*, *b* and *c*) obtained for the color-dose relationship of each combination of radiochromic film (EBT3 or EBT-XD) and portable colorimeter (NixP, NixS, or SpoP) are summarized in Table 1; the parameter values of SpoP were notably different from those of NixP or NixS, as expected from the shapes of the regression curves (Fig. 4).

**Table 1:** Fitting parameter values of the function for regression (Eq. (3): *D* = *a* × Δ*I* + *b* × e^*c*×Δ*I*^)) obtained for the relationships between the black (K) component intensity and X-ray dose of six combinations: two Gafchromic films (EBT3 and EBT-XD) and three portable colorimeters (NixP, NixS and SpoP).

Radiochromic film	Portable colorimeter	Parameter value		
		*a*	*b*	*c*
EBT3	NixP	0.0673	0.00859	0.126
	NixS	0.0676	0.00728	0.143
	SpoP	0.125	0.0179	0.213
EBT-XD	NixP	0.224	0.173	0.114
	NixS	0.200	0.365	0.104
	SpoP	0.333	0.377	0.185

### Angle dependence and dose response

**Figure 5:**
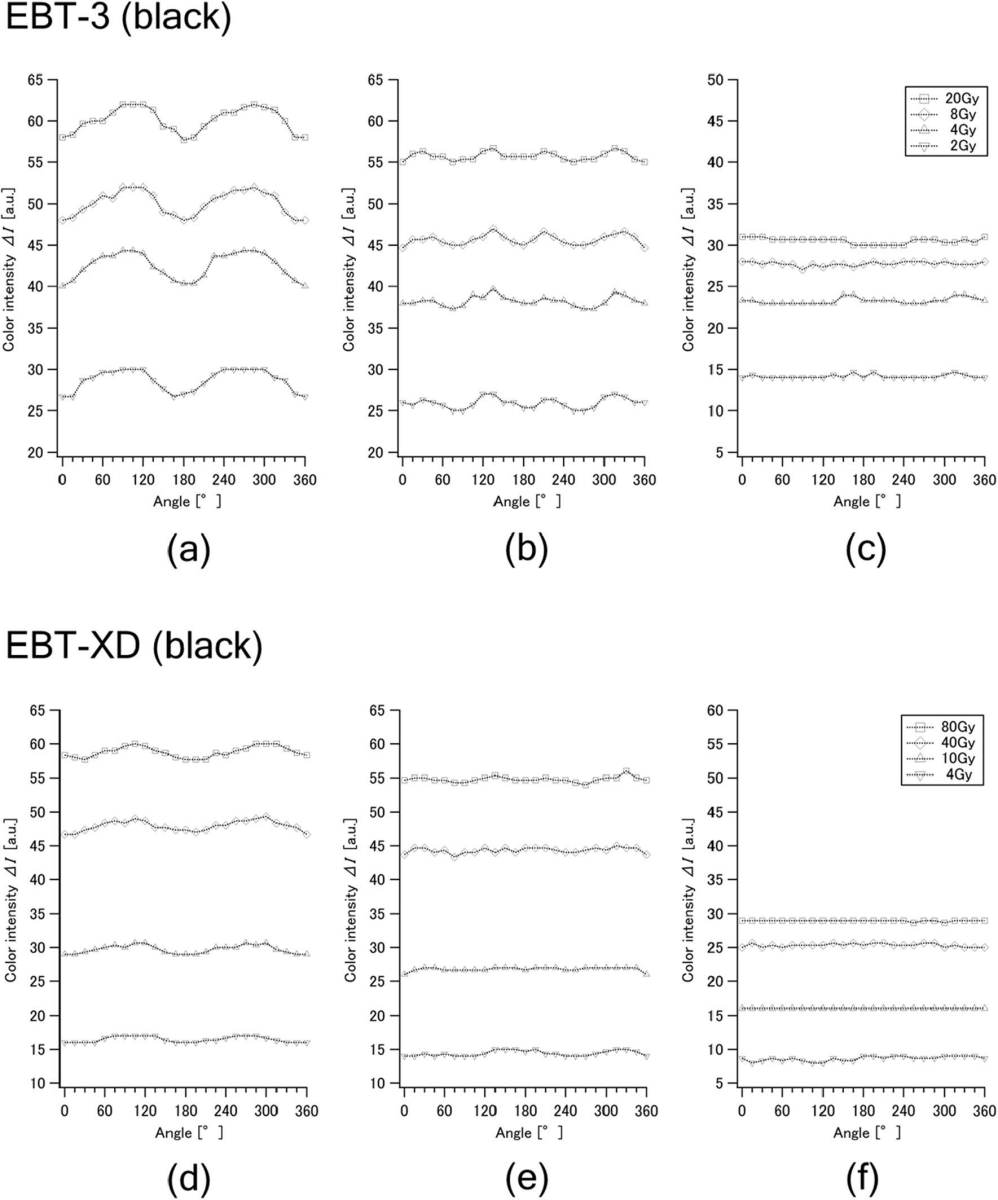
Angle dependences of the black (K) component color intensities of Gafchromic EBT3 measured at 15° intervals with three colorimeters: NixP (**a**, **d**), NixS (**b**, **e**) and SpoP (**c**, **f**) after irradiations with X-rays at 2, 4, 8 and 20 Gy (above) and EBT-XD at 4, 10, 40 and 80 Gy (below). The standard deviation of each plot was less than 3%.

Figure 5 shows the color intensities of the black component measured with three colorimeters rotated at 15° intervals after irradiation with X-rays (160 kV, 6.3 mA) at four dose levels each: 2, 4, 8 and 20 Gy for EBT3 and 4, 10, 40 and 80 Gy for EBT-XD. The standard deviation of each plot was less than 3%. The color intensity (Δ*I*) was calculated with Eq. (2) as the difference in *I*(*D*) measured at each angle and *I*(0) at an angle of 0°.

As seen in the data plots (Fig. 5), the three colorimeters showed considerably different patterns of angle dependence. NixP showed the largest angle dependence, and it decreased in NixS, the same company’s product. Their color intensities showed systematic, wave-like angle-dependent changes with certain cycles: 180° for NixP and 90° for NixS. Good reproducibility was confirmed in the results of NixP, compared to those reported in the previous study^27^. The absolute variation ranges were nearly the same regardless of dose levels; consequently, the relative error became larger at a lower dose level according to the reduced Δ*I*. On the other hand, almost no angle dependence was observed in the SpoP data. It should be noted that the angle-dependent variation of EBT-XD tended to be higher at lower doses (Fig. 5f). The reason for this tendency is unclear at present and needs to be further investigated.

**Figure 6:**
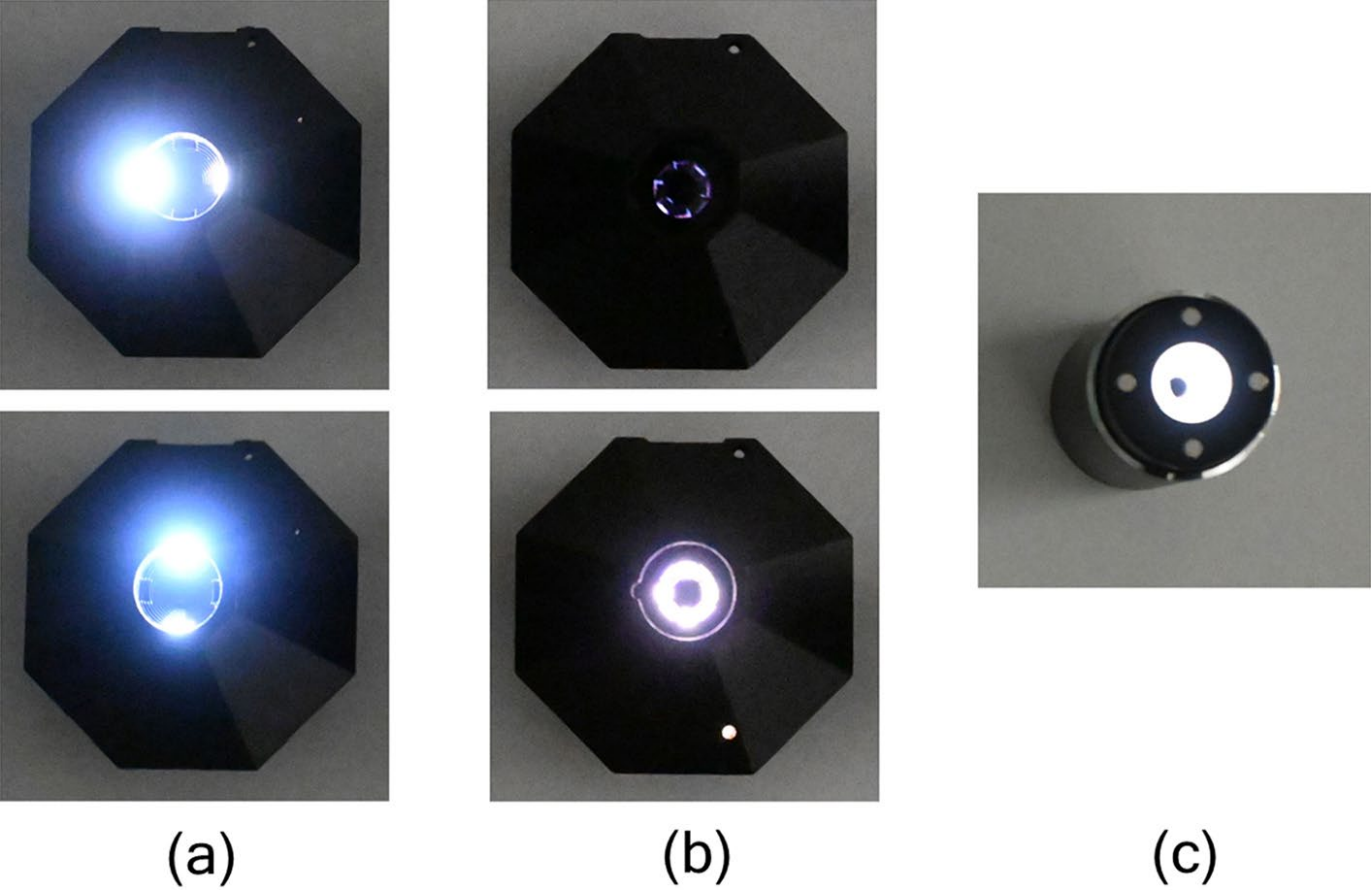
Photographs of the scenes of LED light emissions from three portable colorimeters: (**a**) NixP, (**b**) NixS and (**c**) SpoP.

As the orientation effects (lateral response artifacts) are considered to be caused by the anisotropic light scattering on the film surface when being read with flatbed scanners^7–9,14,31,32^, we assumed that the observed differences in angle dependence among the portable colorimeters (Fig. 5) were attributable to the different patterns of LED light emission for reading films. To confirm this assumption, photographs of the bottom side of each colorimeter were taken in the continuous shooting mode of a digital camera (Z30, Nikon Corp., Tokyo, Japan) during a color reading process, as shown in Fig. 6. As expected, NixP had the smallest number (four) of LET light sources, and NixS had doubled (eight) sources. It was found that SpoP provided a ring-shaped, seamless light emission. These geometric differences in LED light emission sources can explain well the different patterns of observed angle dependences among the three colorimeters (Fig. 5).

According to these findings, it is strongly recommended to pay careful attention to the angle of the colorimeter when reading a film using NixP or NixS. Also, in order to reduce the uncertainty due to a possible irradiation-angle dependence, it is desirable to make sure that the film is attached at the same angle to clothing or protective equipment such as apron and helmet of a subject.

## Conclusion

In the present study, we examined the workability of the recently proposed method for on-site dosimetry by a combination of radiochromic film and portable colorimeter for six combinations of two popular Gafchromic films (EBT3 and EBT-XD) and three commercial portable colorimeters (nix pro2: NixP, nix spectro2: NixS and spectro1 pro: SpoP). While different patterns of dose response and angle dependence were observed, any combination was expected to work for the aim of on-site dosimetry in radiological emergency situations. NixP and NixS would be more suitable for measurements covering a wider dose range than SpoP, while SpoP has an advantageous feature of little angle dependence in film reading, which could simplify the dosimetry process.

The findings presented in this study indicate that we could quickly conduct on-site radiation dosimetry in a simple way by carrying only small devices, i.e., radiochromic films and portable colorimeters. It is expected that the advantageous feature of this novel method would be positively accepted by potential users engaged in the QA of various radiation sources used in medicine, industry and research. For making conclusion about the effectiveness of the proposed method, however, it is required to conduct further investigations on irradiation angle dependence, reproducibility under different conditions/skills, effects of fractionated dose delivery, etc. Also, the authors will continue exploring the potentials of different combinations of radiochromic material and color-reading device to establish the most suitable system for the various real situations of on-site dosimetry.

## Data Availability

The datasets used and/or analysed during the current study are available from the corresponding author upon reasonable request.
